# Differential Transcriptional Effects of EGFR Inhibitors

**DOI:** 10.1371/journal.pone.0102466

**Published:** 2014-09-03

**Authors:** Miroslav Blumenberg

**Affiliations:** The R.O. Perelman Department of Dermatology, Department of Biochemistry and Molecular Pharmacology and the NYU Cancer Institute, NYU Langone Medical Center, New York, New York, United States of America; Wayne State University, United States of America

## Abstract

EGF and its receptor EGFR serve as a paradigm for signaling in cell, molecular and tumor biology. EGFR inhibitors, drugs targeting the intracellular kinase activity and antibodies targeting the extracellular ligand binding, are used to treat breast, lung, colon and other cancers. Nominally affecting the same target, inhibitors have different effects, suggesting that use of inhibitor combinations may provide beneficial in cancer treatment. To explore the specific and the common transcriptional effects of EGFR inhibitors, we present metaanalysis of 20 individual studies comprising 346 microarrays. We identified specific gene subsets regulated by kinase inhibitors, those regulated using antibodies and by suppressing EGFR expression using miR-7. Unreported before, the inhibitors prominently induce lysosome components. All inhibitors rely on related sets of transcription factors and protein kinases, both for transcriptional induction and suppression. However, we find that Gefitinib suppresses apoptosis inhibitors, while inducing cell-cycle inhibitors; conversely, Erlotinib suppresses cell-cycle and cell migration genes, while inducing proapoptotic genes. EGFR-targeting antibodies specifically suppress cell motility, developmental and differentiation processes, while inducing the contractile apparatus. miR-7, distinctively, suppresses cell-cycle genes, while inducing transcription machinery. These metaanalysis results suggest that different inhibitors have overlapping but quite distinct effects in target cells. Judicial use of EGFR-targeting combinations, i.e., simultaneous use of antibodies and multiple kinase inhibitors, may provide more effective cancer treatments with fewer side-effects and avoid development of resistance. We expect, moreover, that specific drug combination treatments can be fine-tuned to achieve specific, personalized results.

## Introduction

Epidermal growth factor, EGF, affects almost all cell types, including eponymous epidermis; its signaling is deregulated in many pathological conditions [1]. EGF and its receptor EGFR constitute, arguably, the most studied model of cellular signaling [2]–[4]. EGFR responsive signaling pathways include GRB2/MAPK, PI3K/AKT, STATs, PLC/PKC, and transcription factors AP1, Myc, Egr1 etc. [1], [2], [4]–[6]. The EGF-regulated genes promote cell-cycle and proliferation, protein synthesis, migration, adhesion, ECM remodeling, angiogenesis, tumorigenesis and metastasis; conversely, apoptosis and terminal differentiation are usually inhibited [7]–[9].

Activated EGFR typifies numerous epithelial malignancies, including cancers of the breast, lung, colon, head-and-neck, pancreas etc. [7]. Therapies that inhibit EGFR became a paradigm for targeted treatment of human cancers and use inhibitors of EGFR kinase, Gefitinib and Erlotinib (a.k.a. Iressa and Tarceva, resp.), or antibodies Lapatinib, Cetuximab, Panitumumab, Zalutumumab, Nimotuzumab and Matuzumab [7], [10]. They can induce tumor regression avoiding some adverse effects of chemotherapy. Drawbacks of EGFR inhibitor therapies are cardiac and renal side-effects, skin toxicity, and intrinsic or acquired resistance to therapy; these limit the duration or dosage of treatment [5], [11].

Whereas all these agents target the same protein, EGFR, different inhibitors use different mechanisms and have different effects [12]. For example, Gefitinib and Erlotinib compete with ATP and inhibit receptor autophosphorylation, retaining effectiveness against constitutively active kinase mutants. Antibodies bind the extracellular domain of receptor, occluding ligand binding, preventing receptor dimerization and activating host immune responses [12], [13].

Many studies used transcriptional profiling to define cellular responses of targeting EGFR. However, the use of different agents, microarray platforms and experimental protocols makes it difficult to characterize the commonalities and the particulars of EGFR inhibition. Our objective here is to use metaanalysis for a comprehensive investigation of transcriptional data. We metaanalysed 20 published transcriptional studies, comprising 346 microarrays, using free, readily available computer programs, e.g., RankProd [14]. We determined the ontological categories overrepresented in the regulated genes and identified potential protein kinases and transcription factors involved.

The results describe large lists of over 2537 suppressed genes and 2251 induced by EGFR inhibitors, with high statistical significance. They identify crucial differences in the genes regulated by antibodies and by kinase inhibitors and specifically the consequences of Gefitinib *vs*. Erlotinib treatments. We also demonstrate the great advantage of metaanalysis over single studies. The work can serve as a paradigm for integration and metaanalysis of transcriptional data in public repositories.

## Methods

To identify transcriptional studies of EGFR inhibition, we searched, PubMed GEO_DataSets [http://www.ncbi.nlm.nih.gov/pubmed] and EMBI-EBI_ArrayExpress [http://www.ebi.ac.uk/arrayexpress], and flagged 50 and 44 data sets, respectively. The results were individually screened yielding 20 studies (Table 1). In 2 of these, GSE17948 and GSE40130, different inhibitors types were used, which were analyzed separately. Thus, we analyzed 22 different sets that compared directly EGFR inhibitor-treated vs. untreated samples. These are listed in Table 1 and described in more detail in Supplement Text S1. Two additional studies were identified: GSE6128 compared inhibitor-treated cells with the same cells after removal of the inhibitor, and GSE17498 used a platform with fewer gene targets; these two studies were not used in our metaanalysis. Most datasets used Affymetrix microarray platforms (11 studies), Illumina and Agilent microarrays were used in 6 and 3 studies, respectively (Table 1). In several studies multiple cell lines or tissues were treated, in these cases each cell line was analyzed separately.

**Table 1:** Data sets used in the metaanalysis.

	Data set	Platform	Set	Control+treated	Target cells, pretreatment	Inhibitor
**1**	**GSE11729**	Affy HG_U133_Plus_2	1	4+12	H1299	gefitinib
			2	12+12	H1299, EGF	gefitinib
			3	2+6	L858R	gefitinib
			4	6+6	L858R, EGF	gefitinib
**2**	**GSE16179**	Affy HG_U133_Plus_2	5	3+3	BT474	lapatinib
			6	3+3	BT474-J4,	lapatinib
			7	3+3	BT474-J4, forteinib	lapatinib
**3**	**GSE20854**	Affy HG_U133_Plus_2	8	4+4	Hec50co	gefiitinib
			9	4+4	Ishikawa H	gefiitinib
**4**	**GSE23206**	Affy HG_U133_Plus_2	10	1+1	H322c	gefiitinib
**5**	**GSE30516**	Affy HG_U133_Plus_2	11	4+9	BT20	erlotinib
			12	3+3	MD	erlotinib
			13	3+3	MCF	erlotinib
**6**	**GSE32975**	Affy HG_U133_Plus_2	14	2+2	HaCat, EGF	gefiitinib
			15	2+2	HaCat, serum	gefiitinib
**7**	**GSE33658**	Affy HG_U133_Plus_2	16	5+6	breast cancer biopsies +/− Gefitinib	gefiitinib
**8**	**GSE6521**	Affy HG_U133_Plus_2	17	2+7	MCF7	AG1478
			18	7+7	MCF7, heregulin	AG1478
**9**	**GSE8141**	Affy HG_U133_Plus_2	19	4+4	MCF7	gefiitinib
			20	4+4	MCF7/HER2-18	gefiitinib
			21	4+4	MCF7/HER2-18, Tamoxifen	gefiitinib
**10**	**GSE19500**	Affy HG_U133A	22	4+4	bronchial epithelial cells	AG1478
			23	3+3	bronchial e.c., high barometric pressure	AG1478
**11**	**GSE17948**	Affy HGU95v2	24	3+4	HMCV-infected monocytes	AG1478
			25	3+4	HMCV-infected monocytes	antiEGFR
**12**	**GSE23428**	Agilent custom array	26	5+5	triple-negative breast cancer biopsies	cetuximab
**13**	**GSE32333**	Agilent-027114	27	3+4	xenografts of A431	nimotuzumab
			28	4+3	xenografts of A431, Rapamicin	nimotuzumab
**14**	**GSE38302**	Agilent-028004	29	1+1	PC-9	gefiitinib
			30	3+3	PC-9GR	gefiitinib
**15**	**GSE38310**	Illumina HumanHT-12 v3.0	31	3+3	HCC827	erlotinib
			32	3+3	HCC827-ER3	erlotinib
			33	3+3	HCC827-T15-2	erlotinib
**16**	**GSE38376**	Illumina HumanHT-12 v3.0	34	3+6	SKBR3	lapatinib
			35	3+6	SKBR3-R	lapatinib
**17**	**GSE40130**	Illumina HumanHT-12 V4.0	36	3+3	FaDu, erlotinib	miR-7
		Illumina HumanWG-6 v3.0	37	2+2	HN5, erlotinib	miR-7
**18**	**GSE19043**	Illumina HumanRef-8 v2	38	3+3	DiFi	gefitinib
			39	3+3	GTL-16	gefitinib
**19**	**GSE23175**	Illumina HumanRef-8 v2	40	1+15	MCF10-A	gefiitinib
			41	1+15	MCF10HER	gefiitinib
**20**	**GSE34557**	Illumina HumanWG-6 v3.0	42	3+3	human epidermal keratinocytes	AG1478
			43	3+3	human epidermal keratinocytes, BMP2	AG1478

Only samples directly comparing EGFR inhibitor-treated with their control samples were used. The comparisons column designates specific sets of treated and control samples. Note that GSE40130 used two different inhibitors, which were analyzed separately. ‘a’ marks the studies used in the ‘Not-Gefitinib’ set.

**Figure 1:**
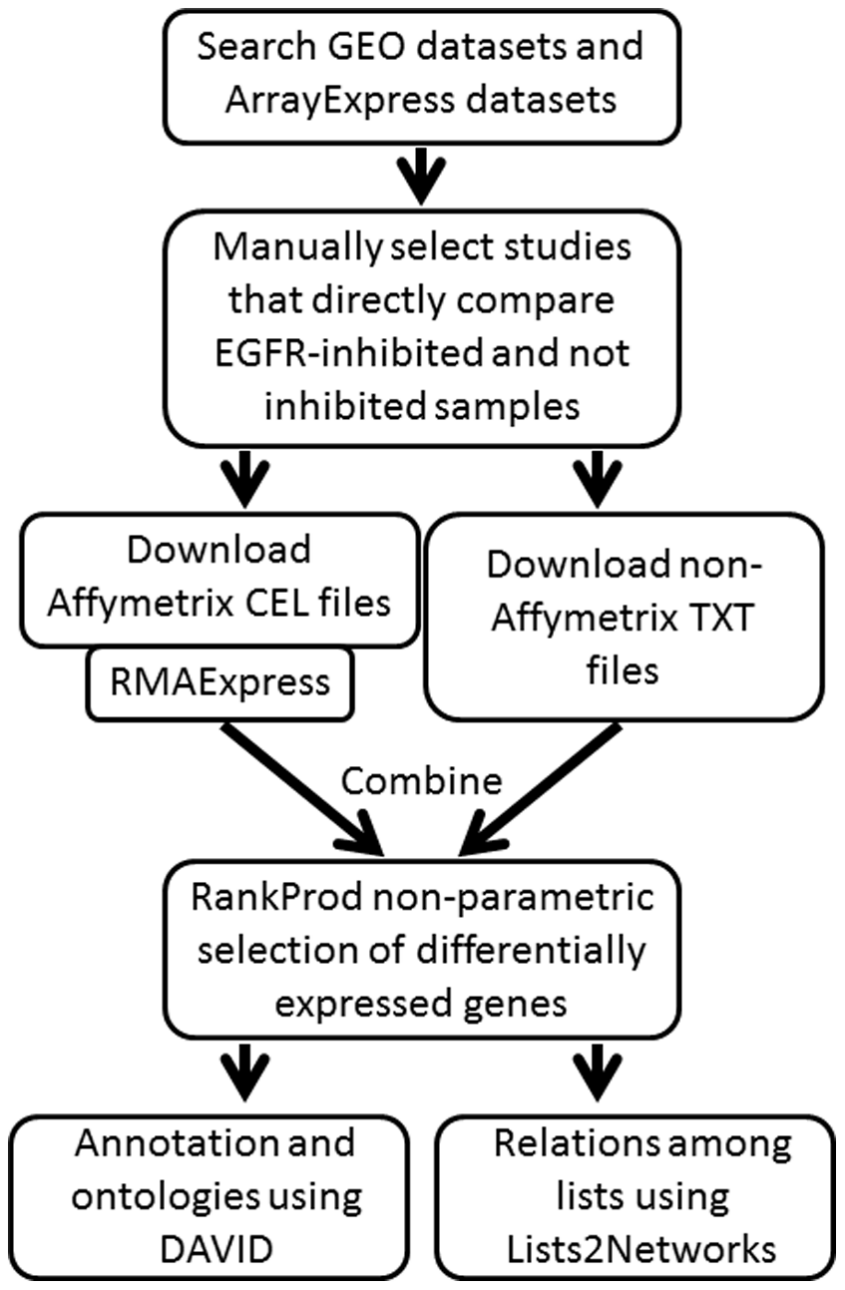
Workflow diagram. The steps used in data assembly and metaanalysis.

We recently published detailed metaanalysis protocols for finding relevant studies in public repositories, downloading data files, microarray quality control, cross-referencing and merging different platforms, dealing with empty cells and negative numbers, selecting data subsets and differentially expressed genes using RankProd, gene lists annotation using the Lists2Networks algorithm, clustering and comparing lists and promoter analysis [15]. The workflow diagram describing meta-profiling and cluster analysis is shown in Fig. 1 Briefly, for the data deposited in studies using the Affymetrix arrays we downloaded CEL files and then processed these using RMAExpress software [16]. To combine data from different platforms we used a non-parametric approach, RankProd, which identifies differentially expressed genes, both up- or down- regulated, based on the estimated percentage of false predictions [14]. Lists of official symbols of regulated genes was submitted to the Lists2Networks analysis program [17]. The program compares lists for mutual overlaps within ontological categories, kinase targets, and disease-associated genes, providing statistical evaluation of the overlaps. For annotation we used the DAVID program suite [18].

## Results

### Identification and characterization of data sets

Because EGFR activation often plays important role in cancers, many teams used various inhibitor agents, microarray platforms and experimental approaches in transcriptional studies of EGFR inhibition. We identified such studies, and then, used RankProd to combine data for metaanalysis. Several studies used multiple cell types, which were compared separately, producing 43 individual pair-wise comparisons (Table 1).

**Figure 2:**
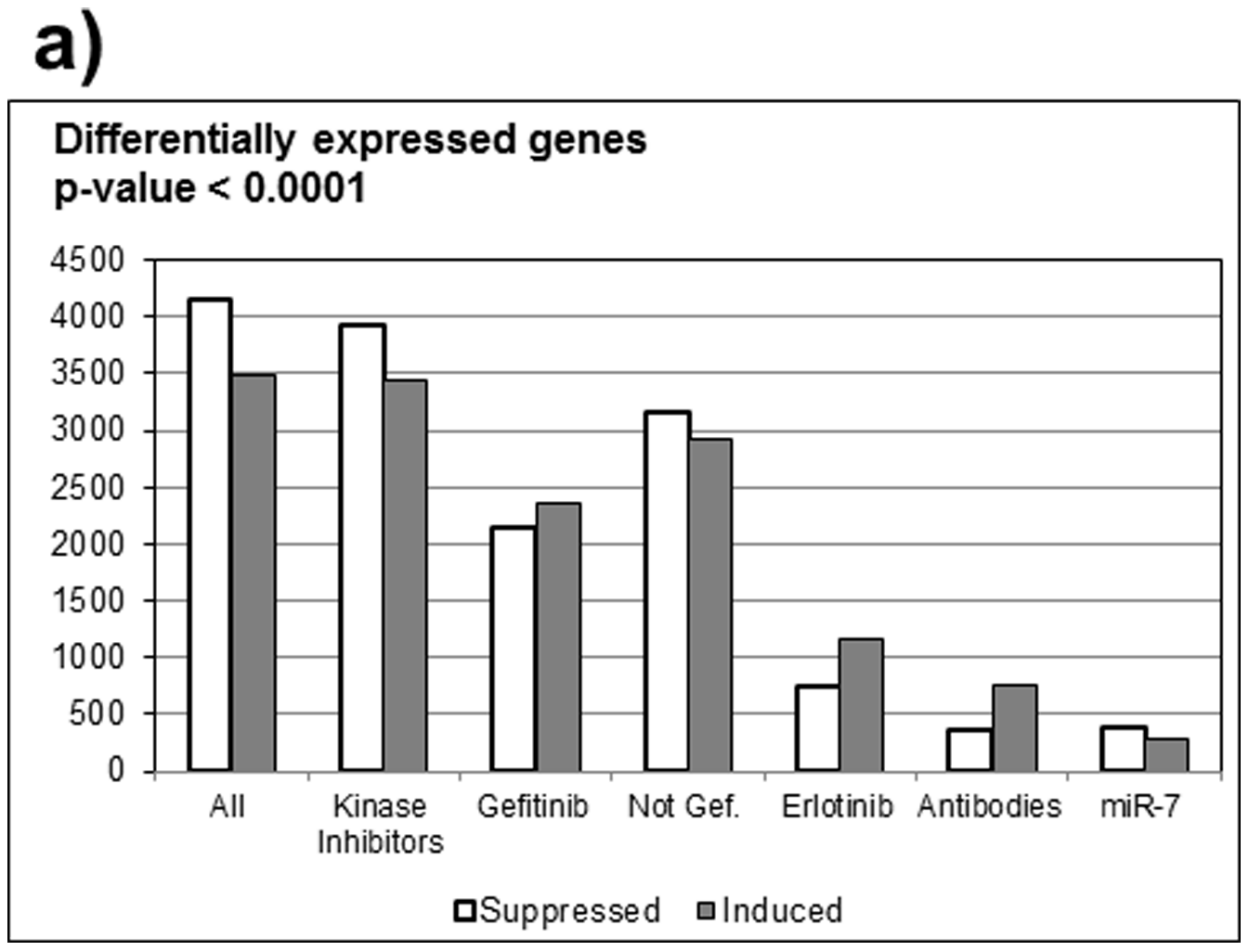
Metaanalysis identifies genes regulated by EGFR inhibitors. Bar graph showing numbers of genes identified as differentially expressed with p-values 10^−4^ or better on different platforms, or using different EGFR inhibitors. Note that for miR-7 p-value 10^−3^ was used as the cut-off because only 10 samples were available.

One of strengths of the metaanalysis approach is its flexibility, which allows direct comparisons of subsets of studies. For example, we selected and separately identified regulated genes in studies using inhibitors of EGFR kinase, ones using antibodies, and one study using miR-7 to suppress EGFR production. The numbers of differentially expressed genes in study subsets are summarized in Fig. 2.

Using DAVID [18], we explored the ontological categories and signaling pathways. The complete annotated list of genes induced and suppressed with p-value better than 10^−4^ from all 346 samples is given in Tables S1, S2. In addition to providing annotations, DAVID estimates which ontological categories and pathways are over-represented in the submitted lists. In Table S3 we provide the top 100 categories in each list, including all 346 samples, and subsets for specific inhibitors. Because DAVID scans many different data bases to identify over-represented categories, the resulting charts contain many redundancies. For example, “cell-cycle”, “cell division”, “mitosis” and “M-phase” are listed as different categories. Such redundancies are largely condensed by ‘clustering’, which combines categories that comprise largely overlapping sets of genes [18]. Therefore, below we focus our analyses on clusters of differentially expressed genes.

### EGFR kinase inhibitors

Several studies used specific kinase inhibitors that compete for binding the ATP pocket of the kinase, Gefitinib, Erlotinib, Lapatinib and Tyrphostin AG1478. While these agents are expected to act by similar mechanism, i.e., inhibiting the kinase enzymatic activity, subtle differences in their effects were seen [12]. The doses of the inhibitors varied among studies, depending on the inhibitor, target cell type etc., but were generally aimed at full, saturating doses that completely inhibit the EGFR kinase. We identified a subset of studies using all kinase inhibitors (i.e., excluding antibodies and miR-7), a subset using Gefitinib, the largest such subdivision, one using all inhibitors except Gefitinib, and one using Erlotinib. We then identified the commonalities and particulars of these subsets and compared them to the complete 346 samples set.

The ontological category ‘nuclear lumen’ is statistically the most prominent one in the suppressed genes (Table 2). Protein modification and metabolism processes, marked ‘P’ in Table 2, are prominent in all suppressed categories, as are transcription and translation, ‘T’. Regulators of apoptosis, both positive and negative, are suppressed by EGFR inhibitors. Interestingly, migration and cell-cycle categories, ‘M’ and ‘C’, did not reach statistical significance in the Gefitinib-suppressed genes, although they are very prominent in the Erlotinib-suppressed genes (Table 2).

**Table 2:** Clusters of ontological categories suppressed by different EGFR kinase inhibitors.

	Suppressed						
	All		Kinase Inhibitors		Gefitinib		Not Gefitinib
	**Enrichment Score: 60.42**		**Enrichment Score: 56.15**		**Enrichment Score: 33.15**		**Enrichment Score: 48.20**
***N***	nuclear lumen	***N***	nuclear lumen	***N***	nuclear lumen	***N***	nuclear lumen
	**Enrichment Score: 17.65**		**Enrichment Score: 16.75**		**Enrichment Score: 7.11**		**Enrichment Score: 15.60**
***T***	ribosome biogenesis	***T***	ribosome biogenesis	***T***	transcription, DNA-dependent	***T***	mRNA processing
	**Enrichment Score: 10.40**		**Enrichment Score: 9.34**		**Enrichment Score: 6.48**		**Enrichment Score: 14.02**
***C***	M phase of mitotic cell cycle	***P***	protein complex assembly	***T***	positive regulation of transcription	***T***	nuclear mRNA splicing, via spliceosome
	**Enrichment Score: 9.89**		**Enrichment Score: 9.20**		**Enrichment Score: 6.29**		**Enrichment Score: 12.72**
***T***	nuclear mRNA splicing, via spliceosome		ATP binding		blood vessel morphogenesis	***P***	protein complex assembly
	**Enrichment Score: 9.87**		**Enrichment Score: 9.19**		**Enrichment Score: 5.87**		**Enrichment Score: 11.87**
	ATP binding	***T***	nuclear mRNA splicing, via spliceosome	***A***	programmed cell death	***C***	M phase of mitotic cell cycle
	**Enrichment Score: 8.58**		**Enrichment Score: 7.61**		**Enrichment Score: 3.93**		**Enrichment Score: 9.22**
***P***	protein complex biogenesis	***C***	M phase of mitotic cell cycle	***P***	regulation of protein kinase activity		ATP binding
	**Enrichment Score: 7.73**		**Enrichment Score: 5.86**		**Enrichment Score: 3.82**		**Enrichment Score: 8.42**
***P***	positive regulation of protein modification process	***A***	programmed cell death	***A***	regulation of apoptosis		mitochondrial inner membrane
	**Enrichment Score: 6.56**		**Enrichment Score: 5.53**		**Enrichment Score: 3.62**		**Enrichment Score: 7.51**
***A***	regulation of apoptosis	***A***	regulation of apoptosis		ATP binding	***P***	regulation of protein ubiquitination
	**Enrichment Score: 6.24**		**Enrichment Score: 5.45**		**Enrichment Score: 3.56**		**Enrichment Score: 6.67**
***A***	programmed cell death	***P***	positive regulation of protein modification process	***P***	protein complex assembly		nucleotide biosynthetic process
	**Enrichment Score: 6.14**		**Enrichment Score: 5.27**		**Enrichment Score: 3.05**		**Enrichment Score: 6.61**
***P***	regulation of protein ubiquitination	***T***	nucleic acid transport		immune system development	***P***	negative regulation of protein metabolism
	**Enrichment Score: 5.43**		**Enrichment Score: 5.16**				**Enrichment Score: 6.27**
***P***	intracellular protein transport	***P***	intracellular protein transport			***T***	RNA transport
	**Enrichment Score: 5.16**		**Enrichment Score: 4.80**				**Enrichment Score: 5.63**
***P***	regulation of protein kinase activity	***T***	DNA/RNA helicase, C-terminal		**Erlotinib**	***P***	intracellular protein transport
	**Enrichment Score: 4.98**		**Enrichment Score: 4.73**		**Enrichment Score: 10.88**		**Enrichment Score: 5.47**
***N***	ATP-dependent helicase activity	***P***	regulation of protein kinase activity	***N***	nuclear lumen	***N***	ATP-dependent helicase activity
	**Enrichment Score: 4.97**		**Enrichment Score: 4.61**		**Enrichment Score: 5.03**		**Enrichment Score: 5.30**
***C***	anaphase-promoting complex-dependent proteasomal ubiquitin-dependent protein catabolic process	***N***	ATP-dependent helicase activity	***P***	regulation of phosphorylation	***A***	regulation of apoptosis
	**Enrichment Score: 4.87**		**Enrichment Score: 4.10**		**Enrichment Score: 3.93**		**Enrichment Score: 4.98**
***T***	RNA transport		endoplasmic reticulum membrane	***A***	regulation of apoptosis	***M***	regulation of cell migration
	**Enrichment Score: 4.63**		**Enrichment Score: 3.79**		**Enrichment Score: 3.88**		**Enrichment Score: 4.54**
***P***	negative regulation of protein modification		blood vessel morphogenesis	***C***	DNA replication initiation	***P***	protein transport
	**Enrichment Score: 4.61**		**Enrichment Score: 3.72**		**Enrichment Score: 2.94**		**Enrichment Score: 4.52**
***M***	regulation of cell migration	***P***	regulation of protein ubiquitination	***C***	DNA unwinding during replication		ribonucleotide biosynthetic process
	**Enrichment Score: 4.37**		**Enrichment Score: 3.64**		**Enrichment Score: 2.80**		**Enrichment Score: 3.87**
***T***	positive regulation of transcription	***T***	positive regulation of transcription	***P***	regulation of protein kinase activity		RNA helicase activity
	**Enrichment Score: 4.26**		**Enrichment Score: 3.58**		**Enrichment Score: 2.36**		**Enrichment Score: 3.53**
***C***	Cyclin, C-terminal	***M***	regulation of cell migration	***M***	regulation of cell migration	***P***	protein import into nucleus
	**Enrichment Score: 4.24**		**Enrichment Score: 3.54**		**Enrichment Score: 2.35**		**Enrichment Score: 3.21**
***A***	positive regulation of apoptosis	***P***	negative regulation of protein metabolic process	***M***	positive regulation of cell migration		nucleoside monophosphate biosynthetsis
	**Enrichment Score: 3.67**		**Enrichment Score: 3.46**		**Enrichment Score: 2.35**		**Enrichment Score: 3.12**
***P***	protein transport	***P***	protein transport	***A***	positive regulation of apoptosis		Chaperonin Cpn60/TCP-1
	**Enrichment Score: 3.63**		**Enrichment Score: 3.34**		**Enrichment Score: 2.29**		
***A***	negative regulation of apoptosis	***C***	Cyclin, N-terminal	***M***	cell migration		
	**Enrichment Score: 3.50**		**Enrichment Score: 3.22**		**Enrichment Score: 2.17**		
***T***	tRNA aminoacylation	***A***	negative regulation of apoptosis	***A***	activation of caspase activity		
	**Enrichment Score: 3.31**		**Enrichment Score: 3.22**		**Enrichment Score: 2.07**		**Legend:**
***M***	positive regulation of cell migration	***T***	Amino acid activation	***C***	lymphocyte proliferation	***A***	**Apoptosis**
	**Enrichment Score: 3.23**		**Enrichment Score: 3.21**		**Enrichment Score: 2.02**	***C***	**Cell cycle**
***P***	peptidyl-asparagine modification		Heat shock protein Hsp70	***A***	negative regulation of apoptosis	***E***	**neural tube**
	**Enrichment Score: 3.22**		**Enrichment Score: 3.14**			***L***	**Lysozyme**
	blood vessel morphogenesis	***A***	positive regulation of apoptosis			***M***	**Migration**
	**Enrichment Score: 3.08**					***N***	**Nucleus**
	Heat shock protein Hsp70					***P***	**Protein modification**
	**Enrichment Score: 3.02**					***S***	**steroid hormone receptor**
***P***	proteolysis					***T***	**Transcription/translation**

For each cluster a representative category was chosen and listed. The enrichment scores are truncated at 2 decimal points. For ease of orientation and discernment, the related categories were marked with a summary letter, as listed in the legend.

The most prominent category induced by EGFR kinase inhibitors is lysosome, ‘L’, the cytoplasmic membrane-bound organelle containing hydrolytic enzymes of intracellular degradation (Table 3). Inhibitors of transcription and translation are induced, as are positive regulators of apoptosis; these effects neatly dovetail with their opposites in the suppressed categories. Interestingly, the cell-cycle inhibitors are induced by Gefitinib: apparently, the mechanisms of shutting down the cell-cycle differ among EGFR kinase inhibitors: while Erlotinib suppresses cell-cycle proteins, Gefitinib induces cell-cycle inhibitors. This interesting observation deserves additional studies because, if confirmed, it could have important implications for cancer treatments.

**Table 3:** Clusters of ontological categories induced by different EGFR kinase inhibitors.

	Induced						
	All		Kinase Inhibitors		Gefitinib		Not Gefitinib
	**Enrichment Score: 7.80**		**Enrichment Score: 6.00**		**Enrichment Score: 5.51**		**Enrichment Score: 9.64**
***L***	lysosome	***L***	lysosome		metal ion binding	***L***	lysosome
	**Enrichment Score: 4.49**		**Enrichment Score: 4.47**		**Enrichment Score: 4.87**		**Enrichment Score: 6.31**
***L***	membrane fraction	***L***	membrane fraction	***T***	negative regulation of transcription	***P***	protein transport
	**Enrichment Score: 4.20**		**Enrichment Score: 4.20**		**Enrichment Score: 3.96**		**Enrichment Score: 4.40**
	metal ion binding	***T***	negative regulation of transcription	***N***	nucleosome assembly	***L***	membrane fraction
	**Enrichment Score: 3.51**		**Enrichment Score: 4.20**		**Enrichment Score: 3.60**		**Enrichment Score: 4.06**
***T***	negative regulation of transcription		metal ion binding	***S***	Steroid hormone receptor	***A***	regulation of apoptosis
	**Enrichment Score: 3.30**		**Enrichment Score: 3.47**		**Enrichment Score: 2.40**		**Enrichment Score: 3.91**
***A***	regulation of apoptosis	***S***	steroid hormone receptor activity	***C***	cyclin-dependent protein kinase inh.	***P***	intracellular protein transport
	**Enrichment Score: 2.86**		**Enrichment Score: 3.06**		**Enrichment Score: 2.38**		**Enrichment Score: 3.42**
***A***	apoptosis	***N***	nucleosome assembly		Ras GTPase	***L***	cytoplasmic membrane-bounded ves.
	**Enrichment Score: 2.82**		**Enrichment Score: 3.02**		**Enrichment Score: 2.34**		**Enrichment Score: 3.34**
***S***	steroid hormone receptor activity		Furin-like cysteine rich region	***E***	cell projection morphogenesis	***T***	negative regulation of transcription
	**Enrichment Score: 2.81**		**Enrichment Score: 2.73**		**Enrichment Score: 2.33**		**Enrichment Score: 3.14**
***E***	primary neural tube formation	***A***	regulation of apoptosis		spermatogenesis	***A***	positive regulation of apoptosis
	**Enrichment Score: 2.79**		**Enrichment Score: 2.47**		**Enrichment Score: 2.32**		**Enrichment Score: 2.85**
***A***	positive regulation of cell death		Ras guanine-nucleotide exch. fact.	***E***	neural tube closure	***A***	programmed cell death
	**Enrichment Score: 2.76**		**Enrichment Score: 2.47**		**Enrichment Score: 2.31**		**Enrichment Score: 2.78**
***E***	cell projection morphogenesis	***L***	cytoplasmic vesicle		BTB/POZ fold	***N***	nucleosome
	**Enrichment Score: 2.59**		**Enrichment Score: 2.36**		**Enrichment Score: 2.29**		**Enrichment Score: 2.45**
	phosphoinositide 3-kinase complex	***E***	primary neural tube formation		Zinc finger, C2H2-type/integrase		small GTPase regulator activity
	**Enrichment Score: 2.54**		**Enrichment Score: 2.28**		**Enrichment Score: 2.28**		**Enrichment Score: 2.31**
***P***	protein transport	***E***	neuron projection development		gamete generation		ATPase activity, transmembrane movement
	**Enrichment Score: 2.39**		**Enrichment Score: 2.20**		**Enrichment Score: 2.03**		**Enrichment Score: 2.08**
	Ras guanine-nucleotide exch. factor	***A***	positive regulation of apoptosis	***L***	membrane fraction		transmembrane receptor tyrosine kinase
	**Enrichment Score: 2.38**		**Enrichment Score: 2.05**		**Enrichment Score: 2.02**		
	Aldehyde dehydrogenase	***P***	intracellular protein transport	***P***	Protein phosphatase 2C		
	**Enrichment Score: 2.35**		**Enrichment Score: 2.04**				
***L***	cytoplasmic membrane-bounded ves.	***P***	protein transport				
	**Enrichment Score: 2.32**		**Enrichment Score: 2.02**		**Erlotinib**		
***N***	nucleosome	***P***	regulation of phosphorylation		**Enrichment Score: 3.52**		
	**Enrichment Score: 2.22**		**Enrichment Score: 2.00**		SH3/SH2 adaptor activity		
***P***	intracellular protein transport	***M***	regulation of cell migration		**Enrichment Score: 2.73**		
	**Enrichment Score: 2.14**			***P***	protein transport		
***T***	negative regulation of DNA binding				**Enrichment Score: 2.64**		
	**Enrichment Score: 2.10**			***N***	nucleosome		
***E***	neuron projection development				**Enrichment Score: 2.53**		
	**Enrichment Score: 2.02**			***A***	programmed cell death		
	Furin-like cysteine rich region				**Enrichment Score: 2.44**		
	**Enrichment Score: 2.00**			***A***	regulation of apoptosis		
***C***	cyclin-dependent protein kinase inhibitor				**Enrichment Score: 2.22**		
					Ras guanine-nucleotide exchange factor		
					**Enrichment Score: 1.83**		
				***A***	induction of apoptosis		

The markings are as in Table 2.

Steroid hormone receptor activity was conspicuous, particularly in the Gefitinib-induced set (Table 3). While its significance is currently unknown, we note the nexus between EGFR inhibition and androgen activity [19], that anti-estrogens are used combined with Gefitinib against breast and lung cancers [20], whereas corticosteroids are used to treat side-effects of Gefitinib [21].

Ontological category ‘neural tube formation’, ‘E’ in Table 3, was also prominent; PubMed search of ‘Neural tube AND Gefitinib’ gave no results.

### Signaling pathways responsive to kinase inhibitors

The kinases signaling downstream from EGFR, especially the MAPK3K family and of course, the EGFR itself, are very prominent, in both suppressed and induced sets (Table S4). Comparing the Gefitinib- and Erlotinib-associated kinases, we see extensive parallels, with the notable exception of GSK3A/GSK3B, present in the Gefitinib, but not Erlotinib list (Table S4). GSK3 is a multifunctional protein controlling glycogen synthase, JUN and other transcription factors, with a role in the WNT and phosphoinositide-3-kinase signaling pathways [22]. PubMed search of Gefitinib & GSK3A yielded no hits, although a nexus between EGFR and the Wnt pathway has been reported [23].

**Figure 3:**
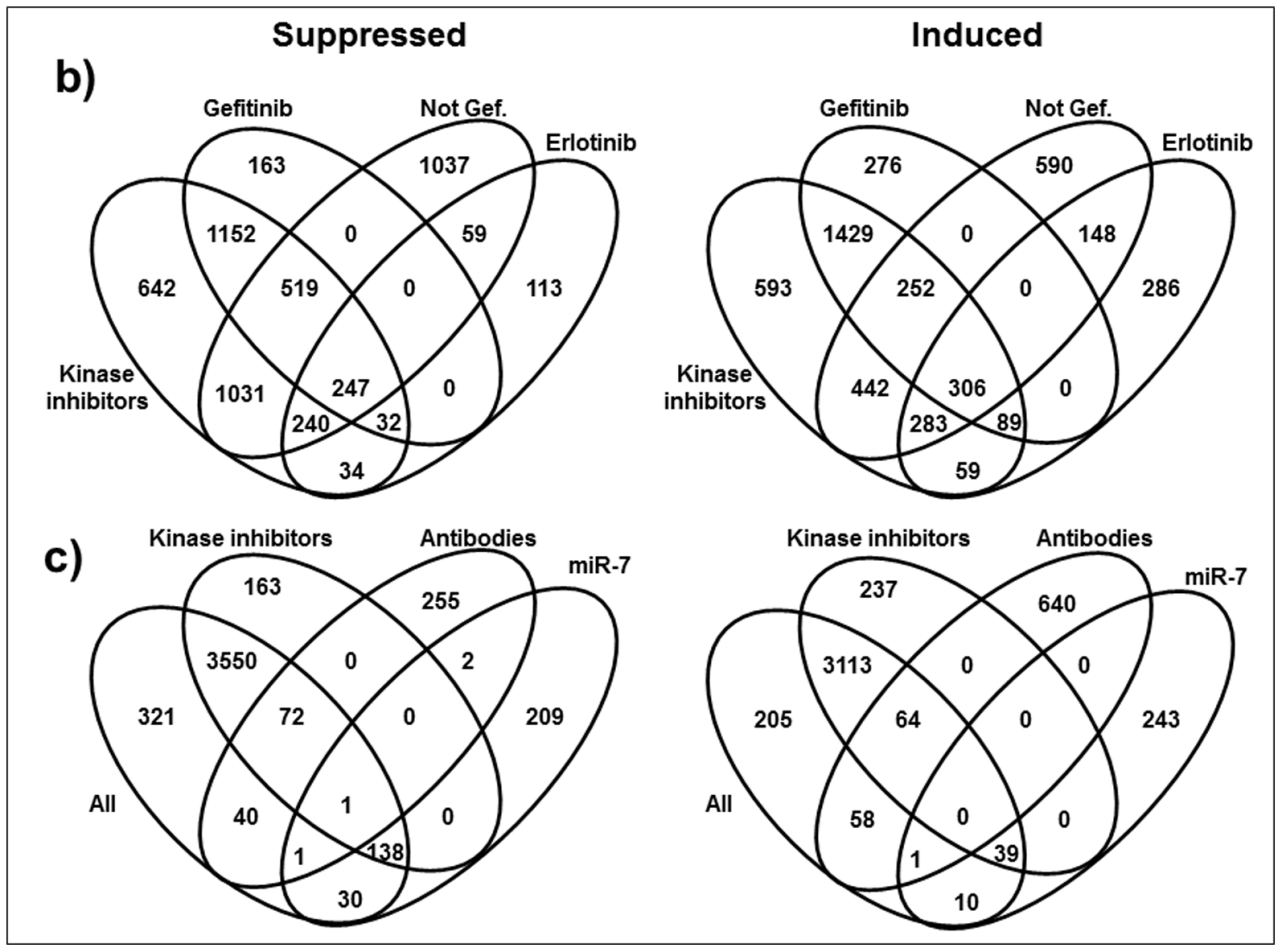
Venn diagrams of genes regulated by the kinase inhibitors. ‘Kinase Inhibitors’ ovals represent genes regulated by all kinase inhibitors, ‘Not Gef.’ those by all inhibitors except Gefitinib. c) Venn diagrams of genes regulated by antibodies and by miR-7. ‘All’ represents the gens from all 346 samples, ‘Kinase Inhibitors’ ovals represent genes regulated by all kinase inhibitors.

We used Venn diagrams to compare overlaps among genes lists (Fig. 3). Genes regulated by individual kinase inhibitors significantly overlap with the genes tagged as regulated by all inhibitors. 306 genes are induced and 247 are suppressed by every kinase inhibitor (Fig. 3). We separately analyzed these genes: all kinase inhibitors suppressed nuclear materiel, translational machinery, protein kinases, cell migration, ECM binding, and regulators of apoptosis; conversely, all inhibitors induced DNA binding proteins, particularly inhibitors of transcription (Table 4).

**Table 4:** Specific clusters of ontological categories regulated by all kinase inhibitors.

	Suppressed 247 genes by all kinase inhibitors				Induced 306 genes		
	Enrichment Score: 12.63	Count	P-Val		Enrichment Score: 3.05	Count	P-Val
	membrane-enclosed lumen.	63	6.E-14		chromatin.	11	4.E-05
***N***	nuclear lumen.	52	4.E-12	***N***	chromosome.	13	3.E-03
	**Enrichment Score: 7.21**				**Enrichment Score: 2.48**		
	rRNA processing.	12	1.E-08		Histone core.	7	8.E-06
***T***	rRNA metabolic process.	12	2.E-08	***N***	nucleosome.	6	5.E-04
	**Enrichment Score: 4.88**				**Enrichment Score: 2.40**		
	regulation of phosphate metabolic process.	20	7.E-06		negative regulation of macromol. biosynth. process.	16	1.E-03
***P***	regulation of phosphorylation.	18	5.E-05	***T***	negative regulation of gene expression.	14	4.E-03
	**Enrichment Score: 4.02**						
	regulation of phosphorylation.	18	5.E-05				
***P***	regulation of protein kinase activity.	15	8.E-05				
	**Enrichment Score: 2.51**						
	regulation of cell motion.	10	6.E-04				
***M***	positive regulation of cell migration.	6	5.E-03				
	**Enrichment Score: 2.42**						
	activation of caspase activity.	6	5.E-04				
***A***	positive regulation of caspase activity.	6	7.E-04				
	**Enrichment Score: 2.13**						
	Sprouty.	4	5.E-05				
***A***	negative regulation of MAP kinase activity.	5	9.E-04				
	**Enrichment Score: 2.02**						
	regulation of apoptosis.	19	9.E-03				
	regulation of programmed cell death.	19	1.E-02				
	**Enrichment Score: 2.01**						
	extracellular matrix binding.	4	4.E-03				
	laminin binding.	3	7.E-03				
	**Enrichment Score: 2.00**						
	apoptosis.	16	6.E-03				
***A***	programmed cell death.	16	7.E-03				

The data pertain to the intersection of all lists in Fig. 3. The presentation is as in Table 2, except we kept 2 characteristic representative categories for each cluster. Enrichment score of 2.0 was used as the cut-off.

We analyzed separately the sets of genes responding to Gefitinib, Erlotinib, and the non-Gefitinib inhibitors (Fig. 3, Table S5). A caveat in this analysis is that differentially reaching statistical significance does not necessarily mean differential transcriptional responses. Negative regulators of transcription are suppressed by Gefitinib (Table S5). This is surprising because in parallel to other kinase inhibitors, Gefitinib suppresses positive regulators of transcription (Tables 2, 3). We hypothesize that Gefitinib induces very specific transcriptional responses, over a background of general inhibition of transcription. The transcription/translation machinery is, as expected, suppressed by the non-Gefitinib inhibitors, while inhibitors of protein transport, lysosomal degradation and transcription are induced (Table S5).

Unanticipated, Gefitinib induces the cell-cycle machinery (Table S5). This is an unexpected response to EGFR inhibition and we note that Gefitinib, unlike Erlotinib and other kinase inhibitors, does not generally suppress cell-cycle genes (Tables 2, 3). Confirming the above, the non-Gefitinib kinase inhibitors (which include Erlotinib) specifically suppressed the cell-cycle machinery (Table S5). This observation reinforces the hypothesis that Gefitinib, specifically among EGFR kinase inhibitors, may not directly inhibit the cell-cycle. Obviously, such contentions need direct lab-bench proof.

The transcriptional changes in response to EGFR inhibition reflect, presumably, the changes in the activity of transcription factors. We identified the transcription factors with binding sites statistically overrepresented in the regulated genes. In general, very similar sets of transcription factors appear activated by different receptors. There is an overlap between the transcription factors responsible for the induced genes and for the suppressed ones (Table S6).

### Antibody inhibitors targeting EGFR

Antibodies inhibit EGFR by binding to the extracellular domain of EGFR, occluding the ligand binding site and preventing receptor dimerization. Treatment with antibodies is plagued by resistance from mutations that constitutively activate the kinase. To analyze the transcriptional effects caused by EGFR-targeting antibodies, we culled from the total only GSE32975, GSE17948, GSE23468 and GSE323333, 39 samples in total (Table 1). Because the number of studies and microarrays using antibodies is smaller, the ‘enrichment scores’ for the ontological categories are lower too.

Specifically, antibodies suppress genes associated with cell migration (Table 5). EGFR signaling is involved in actin cytoskeleton reorganization; however, the antibody inhibitors, but not kinase inhibitors, specifically induce the cytoskeletal contractile elements dominated by myosin genes (marked with tildes in Table 5). At present we have no explanation why the antibody inhibitors preferentially regulate the contractile apparatus. Antibodies do not suppress cell-cycle or apoptosis associated genes, which are prominently suppressed by kinase inhibitors (c.f. Table 2).

**Table 5:** Clusters of ontological categories regulated by antibodies targeting EGFR.

	Antibodies			By antibodies only			Both antibodies and kinase inhibitors	
	Suppressed			255 genes			112 genes	
	Enrichment Score: 9.80	p-Val		Enrichment Score: 7.39	p-Val		Enrichment Score: 3.85	p-Val
	intermediate filament cytoskeleton.	1.E-08		Intermediate filament protein,	2.E-13		response to steroid hormone stimulus.	1.E-05
	**Enrichment Score: 4.94**		**	keratin filament.	5.E-08	***S***	response to hormone stimulus.	1.E-04
	response to hormone stimulus.	1.E-06		**Enrichment Score: 4.48**			**Enrichment Score: 3.08**	
	**Enrichment Score: 4.08**			regulation of hair cycle.	8.E-06		regulation of cell migration.	6.E-04
	regulation of hair follicle development.	2.E-05	**	regulation of epidermis development.	6.E-04	***M***	regulation of locomotion.	1.E-03
	**Enrichment Score: 3.52**			**Enrichment Score: 3.71**			**Enrichment Score: 2.45**	
	hemopoiesis.	2.E-04		female gonad development.	1.E-04		hemopoiesis.	2.E-03
	**Enrichment Score: 3.24**		**	sex differentiation.	1.E-04		immune system development.	5.E-03
***P***	regulation of phosphorylation.	1.E-03		**Enrichment Score: 3.47**			**Enrichment Score: 2.42**	
	**Enrichment Score: 3.22**			ovulation cycle process.	1.E-04		extracellular matrix binding.	2.E-04
***M***	regulation of cell migration.	3.E-04	**	ovulation cycle.	1.E-04		integrin binding.	2.E-03
	**Enrichment Score: 3.11**			**Enrichment Score: 3.47**			**Enrichment Score: 2.34**	
***M***	Cell structure and motility.	6.E-03		hair follicle development.	3.E-04		regulation, phosphate metabolic process.	3.E-03
	**Enrichment Score: 3.02**		**	molting cycle process.	3.E-04	***P***	regulation of phosphorylation.	1.E-02
	female gonad development.	5.E-04		**Enrichment Score: 3.42**			**Enrichment Score: 2.15**	
	**Enrichment Score: 3.01**			response to hormone stimulus.	9.E-05		positive regulation of cell migration.	5.E-03
***M***	cell migration.	5.E-04		response to endogenous stimulus.	2.E-04	***M***	positive regulation of cell motion.	7.E-03
	**Enrichment Score: 2.99**			**Enrichment Score: 3.23**			**Enrichment Score: 2.13**	
	hair cycle process.	9.E-04		reproductive process.	3.E-04		Intermediate filament protein.	2.E-03
			**	gamete generation.	3.E-03		Type II keratin.	4.E-03
				**Enrichment Score: 2.51**				
				hemopoiesis.	2.E-03			
			**	immune system development.	5.E-03			
				**Enrichment Score: 2.14**				
				cell migration.	5.E-03			
			M	cell motility.	8.E-03			
				**Enrichment Score: 2.08**				
				blood vessel morphogenesis.	5.E-03			
			**	vasculature development.	1.E-02			
				**Enrichment Score: 2.04**				
				erythrocyte differentiation.	4.E-03			
			**	erythrocyte homeostasis.	6.E-03			
	**Induced**			**640 genes**			**122 genes**	
	**Enrichment Score: 10.27**			**Enrichment Score: 10.03**			**Enrichment Score: 1.44**	
	contractile fiber.	3.E-12		contractile fiber.	5.E-12		tissue regeneration.	1.E-02
	**Enrichment Score: 4.98**		***∼∼***	myofibril.	1.E-10		regeneration.	4.E-02
	glycosaminoglycan binding.	3.E-04		**Enrichment Score: 5.57**			**Enrichment Score: 1.40**	
	**Enrichment Score: 2.53**			polysaccharide binding.	4.E-07		apoptosis.	7.E-02
	myosin filament.	8.E-04		glycosaminoglycan binding.	1.E-04	***A***	programmed cell death.	7.E-02
	**Enrichment Score: 2.41**			**Enrichment Score: 5.12**			**Enrichment Score: 1.24**	
	lung development.	5.E-03		muscle myosin complex.	2.E-06		response to nutrient.	3.E-02
	**Enrichment Score: 2.14**		***∼∼***	myosin II complex.	4.E-06		response to extracellular stimulus.	9.E-02
***E***	neural crest cell differentiation.	5.E-03		**Enrichment Score: 3.64**				
	**Enrichment Score: 2.05**			locomotory behavior.	2.E-04			
	skeletal muscle contraction.	2.E-03	***M***	chemotaxis.	2.E-04			
	**Enrichment Score: 1.96**			**Enrichment Score: 3.64**				
	myofibril assembly.	9.E-03		blood vessel development.	6.E-05			
	**Enrichment Score: 1.86**			vasculature development.	8.E-05			
***M***	cell migration.	1.E-02		**Enrichment Score: 2.52**				
				Myosin tail.	1.E-05			
			***∼∼***	Heavy chain of myosin.	9.E-04			
				**Enrichment Score: 2.19**				
				anterior/posterior pattern formation.	5.E-03			
				pattern specification process.	1.E-02			
				**Enrichment Score: 2.17**				
				skeletal muscle contraction.	1.E-03			
			***∼∼***	troponin complex.	1.E-02			
				**Enrichment Score: 2.10**				
				myofibril assembly.	7.E-03			
			***∼∼***	actomyosin structure organization.	2.E-02			

Panels pertain to the 255 and 640 genes specifically regulated by the antibodies, and to 72+40 and 64+58 genes shared by the antibodies and kinase inhibitors (see Fig. 3). Asterisks mark the ontological categories related to tissue development, tildes the categories related to contractility, specifically induced by the antibodies. The letter markings are as in the legend of Table 2.

Importantly, none of the protein kinases or transcription factors reached statistical significance in association with antibody-regulated genes (Table S4). Comparing overlaps among differentially regulated genes, we find 255 genes specifically suppressed by the antibodies, while 112 (40+72) are suppressed in common with the kinase inhibitors (Fig. 3). Focusing on these two sets, we find that the ontological categories suppressed only by the antibodies are associated with developmental processes, specifically with a) epidermis/hair, b) reproduction/ovulation and c) vasculature/blood development (asterisks in Table 5). These results are quite unexpected and point, on one hand, to serious side-effects of EGFR-targeting therapies and on the other underline the importance of EGFR signaling in the homeostasis of these self-renewing tissues.

The suppressed processes in common with the kinase inhibitors comprise migration and responses to steroid hormone; the induced ones include apoptosis regulation, but, importantly, we caution that the enrichment scores are low, which precludes making confident conclusions (Table 5).

### Effects of miR-7

miR-7 suppresses EGFR production by direct interaction with two sites within the EGFR mRNA [24]. Pleiotropic, miR-7 inhibits multiple targets downstream from and in addition to EGFR signaling, including Akt, ERK1/2, RAF1, IRS1&2 and PAK1.

Predictably, the effects of miR-7 differ significantly from those of other agents. Ontological categories suppressed by miR-7 notably include cell-cycle (Table 6). Ribosome assembly and biogenesis is, however, induced by miR-7 (Table 6). The MAPK3K family targets the miR-7 regulated genes; MAP3K14 and RPK3 specifically the miR-7 induced genes. However, because miR-7 inhibits multiple targets, it is currently not possible to assign its effects specifically to the EGFR-suppressing function.

**Table 6:** Clusters of ontological categories regulated by miR-7. Note that the cell-cycle related clusters are suppressed by miR-7, while the transcriptional machinery is induced.

	Suppressed by miR-7			By miR-7 only	
	Enrichment Score: 8.97	p-Val		Enrichment Score: 2.48	p-Val
***C***	mitotic cell cycle.	3.E-11		mitosis.	3.E-03
	**Enrichment Score: 8.06**		***C***	M phase of mitotic cell cycle.	3.E-03
***C***	M phase of mitotic cell cycle.	1.E-09		**Enrichment Score: 2.23**	
	**Enrichment Score: 1.71**			centrosome.	2.E-03
***N***	nuclear lumen.	4.E-02	***C***	microtubule organizing center.	4.E-03
	**Enrichment Score: 1.56**			**Enrichment Score: 1.83**	
***C***	mitotic sister chromatid segregation.	6.E-03		negative regulation of protein metabolism.	7.E-03
	**Enrichment Score: 1.42**		***P***	regulation of protein metabolic process.	8.E-02
	Heat shock protein DnaJ.	2.E-02		**Enrichment Score: 1.37**	
				response to endogenous stimulus.	1.E-02
			***S***	response to hormone stimulus.	3.E-02
	**Induced**				
	**Enrichment Score: 16.22**	**p-Val**		**Enrichment Score: 17.26**	**p-Val**
***T***	structural constituent of ribosome.	3.E-18		translational elongation.	3.E-24
	**Enrichment Score: 15.82**		***T***	Ribosome.	1.E-18
***T***	cytosolic ribosome.	4.E-18		**Enrichment Score: 15.21**	
	**Enrichment Score: 3.35**			cytosolic ribosome.	2.E-17
***T***	rRNA processing.	1.E-04	***T***	ribosomal subunit.	9.E-16
	**Enrichment Score: 2.61**			**Enrichment Score: 3.75**	
	mitochondrial inner membrane.	2.E-04		rRNA processing.	4.E-05
	**Enrichment Score: 2.40**		***T***	ribosome biogenesis.	2.E-04
	Ubiquitin.	5.E-03		**Enrichment Score: 2.99**	
	**Enrichment Score: 1.94**			mitochondrial inner membrane.	8.E-05
	intermediate filament cytoskeleton.	7.E-02		mitochondrial envelope.	1.E-03
	**Enrichment Score: 1.93**			**Enrichment Score: 2.59**	
	mitochondrial ATP synthase.	5.E-04		Ubiquitin conserved site.	1.E-03
	**Enrichment Score: 1.78**		***P***	Ubiquitin.	3.E-03
	mitochondrial lumen.	1.E-01		**Enrichment Score: 2.13**	
				mitochondrial ATP synthase complex.	3.E-04
				proton-transporting ATP synthase.	5.E-04

## Discussion

This work demonstrates the advantage of metaanalysis over single studies: metaanalysis provided 5 times more regulated genes than the largest single study. Importantly, coherent, single platform metaanalysis has advantages over an assortment of platforms, but in general analyses of large data sets provide more regulated genes than of smaller ones. Because we used free, publically available metaanalysis programs, this work can serve as a paradigm for integration and metaanalysis of transcriptional data in public repositories (Fig. 1).

Large lists of regulated genes allowed us to identify novel ontological categories affected by EGFR inhibition. As expected, the suppressed genes are associated with cell-cycle, migration, transcription and protein synthesis, while the induced genes include ones associated with apoptosis, and inhibition of transcription and translation. Unexpectedly, the induced categories also include genes associated with lysosome and with steroid hormone receptor activity. The induction of lysosomal genes by EGFR inhibition is a component of the autophagy, a process commonly associated with EGFR inhibition [25]. The induction of lysosomal genes may also play a significant role in, e.g., reducing the effectiveness of the inhibitors by degradation.

Separate analyses of kinase inhibitors and antibodies identified important differences and commonalities. For example, antibodies suppress cell migration genes, much less the cell-cycle genes, while the reverse is true for kinase inhibitors. The differences do not derive from differences in cell types targeted, i.e., muscle or neuronal, they seem to be specific consequences of using different agents to inhibit EGFR. The molecular mechanisms causing these differences are not known; we speculate that antibodies, being large molecules, perturb the agglomeration of EGFR in the cell membrane [26]. This may affects the interaction between EGFR and other membrane-bound proteins transducing some of the downstream signals.

Our results fit well with previous findings that combination treatments with multiple agents can have synergistic effects [13]. They suggest that combination treatments targeting EGFR, i.e., simultaneous use of antibodies and kinase inhibitors, may be beneficial for avoiding development of resistance. Moreover, we suggest that using specific combinations of agents can be fine-tuned and personalized to achieve patient-specific treatment responses. For example, we speculate that highly proliferative but rarely metastasizing cancers may benefit more from treatment with Erlotinib, which strongly affects cell cycle progression genes (Table 2), whereas highly metastatic tumors may benefit more from antibody therapies, which strongly affect cell motility (Table 5). Gefitinib seems less proapoptotic than other kinase inhibitors, which may reduce side effects of targeting EGFR in specific cases (Table 3). These are just guidelines, which will need experimental corroboration.

Antibodies specifically suppress the developmental effects of EGFR (e.g., in skin, hair, vasculature and gonads). Antibodies also specifically induce genes associated with the contractile apparatus. Such effects perhaps depend on altered EGFR-containing multi-protein complex formation in cell membrane, a cytoskeleton-dependent process [4], [26].

While kinase inhibitor drugs are supposed to act with same mechanisms, there are characteristic differences in on-off rates, receptor conformation and accessibilities of its serine/threonine/tyrosine target substrates [27]. Apparently, individual inhibitors use distinct mechanisms to achieve similar results: cell-cycle inhibitors are induced by Gefitinib, conversely cell-cycle promoters are suppressed by Erlotinib – the same results achieved by different routes. Targets of GSK3 are significantly suppressed by Gefitinib, not by other agents. Neural tube closure and steroid hormone receptor activity are particular targets of induction by Gefitinib, which deserves further studies. These results may suggest specific preference for use of Gefitinib in certain tumors, e.g., glioblastomas, and more problematic side-effects in other tumors.

The signal transducing kinases inhibited by EGFR-targeting agents largely overlap. They include known members of the EGFR signaling cascade, MAP3K being the most prominent. Although individual agents are associated with individual kinases, the differences seem subtle and it is unclear presently whether this is due to statistical effects of different study sizes, i.e., numbers of samples, or real mechanistic differences responding to different agents. Even more homogenous are the transcription factors that, presumably, control the expression of the regulated genes: similar sets of TFs respond to multiple EGFR inhibitors, although there are differences in their relative orders and p-values. The significance of these subtle differences is at present dubious. Highly overlapping sets of TFs regulate both the suppressed and the induced genes. This would suggest that constellations of TFs in the promoters of regulated genes, their interactions with auxiliary proteins or as yet unknown TFs determine whether a gene is induced or suppressed by the inhibition of EGFR.

## Supporting Information

Table S1 **The 2537 genes suppressed by the EGFR inhibitors.** The Affymetrix ID, gene name, cytoband, Entrez ID, associated KEGG pathway, official symbol and OMIM disease associations are included.
(XLSX)

Table S2 **The 2251 genes induced by the EGFR inhibitors.**
(XLSX)

Table S3 **Ontological categories.** Top 100 ontological categories, by p-values, are listed for each list of genes mentioned in the text.
(XLSX)

Table S4 **Kinases associated with regulated genes.** The MAPK3 family kinases participate in both induction and suppression of genes by EGFR inhibitors; they are particularly important in the induction by miR-7. Note also the differences between the kinases responsive to Gefitinib and those responsive to Erlotinib, such as GSK3s. No kinase reached statistical significance in the analysis of genes regulated by antibodies.
(XLSX)

Table S5 **Clusters of ontology categories specifically regulated by individual kinase inhibitors.** Note the much relaxed enrichment scores used as cut-offs because of relatively short lists of genes.
(XLSX)

Table S6 **Top 30 transcription factors associated with the regulated genes.** Grey marks the top 10 factors in analysis of all 346 samples; the same 10 are marked with grey in the individual analyses as well. a) Suppressed genes. b) Induced genes. We find that 8 out of 10 top transcription factors regulate both the suppressed and the induced genes, these are marked with asterisks. The transcription factors associated with the miR-7 regulated genes and with genes suppressed by the antibodies did not reach statistical significance.
(XLSX)

Text S1 **Description of studies used in this metaanalysis.**
(DOCX)
