# Supplementary material for: Differential Transcriptional Effects of EGFR Inhibitors
Source: PLoS One. 2014 Sep 3;9(9):e102466. doi: 10.1371/journal.pone.0102466 (PMC4153546; doi:10.1371/journal.pone.0102466)
Supplement: Text S1 — Description of studies used in this metaanalysis. (DOCX) [file pone.0102466.s007.docx]

**GSE11729** comprises 60 microarrays, analysis of cell line H1299, human non-small cell lung cancer cells, and a derivative line with the EGFR activating mutation L858R +/- Gefitinib both in the presence and absence of EGF. Time courses to 10h were followed [[1](#_ENREF_1)]. We separately analyzed 4 sets: H1299 +/- Gefitinib, H1299 +EGF +/- Gefitinib, H1299L858R +/- Gefitinib, H1299L858R +EGF +/- Gefitinib.

**GSE16179** comprises 18 microarrays, analysis of cell line BT474 and its lapatinib-resistant variant, BT474-J4 +/- lapatinib and BT474-J4 + foretinib (a cMet inhibitor) +/- lapatinib, all in triplicates. We separately analyzed 3 sets: BT474 +/- lapatinib, BT474-J4 +/- lapatinib, BT474-J4 + forteinib +/- lapatinib [[2](#_ENREF_2)].

**GSE20854** comprises 24 microarrays, analysis of 2 cell lines Hec50co and Ishikawa H +/- EGF or +/- Gefitinib. We separately analyzed the 2 cell lines +/- Gefitinib, ignoring the EGF-treated, and combining the 12 and 24h time points [[3](#_ENREF_3)].

**GSE23206** comprises 2 microarrays, analysis of H322c cells +/- Gefitinib [[4](#_ENREF_4)].

**GSE30516** comprises 25 microarrays, analysis of BT20, MD and MCF cells +/- Erlotinib. We separately analyzed the 3 cell types, but combined the 30 min, 1 and 24h time points for BT20 cells [[5](#_ENREF_5)].

**GSE32975** comprises 58 microarrays, analysis of HaCat cells transfected with a vector expression EGFR and treated with several inhibitors. Because we are specifically interested in the effects of Gefitinib only, we separately analyzed 2 sets, cells treated with EGF +/- Gefitinib or with serum +/- Gefitinib, combining the 4 and 8 h time points [[6](#_ENREF_6)].

**GSE33658** comprises 22 microarrays, analysis of breast cancer biopsies, post treatment with anastrozole and fulvestrant +/- Gefitinib. We did not analyze the pre-treatment biopsies [[7](#_ENREF_7)].

**GSE6521** comprises 37 microarrays, analysis of MCF7 cell line treated or not with heregulin +/- AG1478 (and other, drugs not relevant for this study). We separately analyzed 2 sets, cells +/- AG1478 or with heregulin +/- AG1478, combining the 5 to 90 min time points.

**GSE8141** comprises 65 microarrays, analysis of subcutaneous xenografts of MCF7 and MCF7/HER2-18 cells on athymic mice, comparing the effects of estrogen, estrogen withdrawal, and tamoxifen, all with or without Gefitinib [[8](#_ENREF_8)]. Because we are interested specifically in the effects of Gefitinib, we separately compared 3 groups: MCF7 +/- Gefitinib (4+2 microarrays), MCF7/HER2-18 +/- Gefitinib (4+4 microarrays) and MCF7/HER2-18 with Tamoxifen +/- Gefitinib (5+4 microarrays), for a total of 23 arrays.

**GSE19500** comprises 17 microarrays, analysis of bronchial epithelial cells in normal and high barometric pressure +/- AG1478. We separately analyzed 2 sets, cells +/- AG1478 or pressurized +/- AG1478, combining the 1, 3 and 8h time points [[9](#_ENREF_9)].

**GSE17948** comprises 16 microarrays, analysis of HMCV-infected monocytes, treated with either AG1478, or an EGFR-blocking antibody. We compared separately the AG1478 and the antibody-treated cells with the HMCV-infected monocytes and ignored the mock-infected controls [[10](#_ENREF_10)].

**GSE23428** comprises 36 microarrays, analysis of triple-negative breast cancer biopsies before and after treatment with Cetuximab (samples treated with combination of Cetuximab + carboplatin were not included) [[11](#_ENREF_11)].

**GSE32333** comprises 14 microarrays, analysis of subcutaneous xenografts of A431 cells treated with Rapamicin +/- Nimotuzumab. We separately compared control +/- Nimotuzumab (3+4 arrays) and Rapamicin-treated +/- Nimotuzumab (3+4 arrays).

**GSE38302** comprises 8 microarrays, analysis of Gefitinib-sensitive cell line PC-9 +/- Gefitinib (1+1 array) and resistant cell line PC-9GR +/- Gefitinib (3+3 arrays). Only the 2 arrays with samples from sensitive cells were used [[12](#_ENREF_12)].

**GSE38310** comprises 18 microarrays, analysis of 3 cell lines, HCC827 and its resistant derivatives, ER3 and T15-2. All 3 were treated with Erlotinib. We separately analyzed the 3 cell types [[13](#_ENREF_13)].

**GSE38376** comprises 18 microarrays, analysis of SKBR3 cell line and its resistant derivative, SKBR3-R, treated with two doses of Lapatinib. We separately analyzed the sensitive and the resistant cell types [[14](#_ENREF_14)].

**GSE40130** comprises 10 microarrays, analysis of FaDu and HN5 cell lines transfected with an miR-7 expressing construct; miR-7 targets EGFR expression. The two call lines were analyzed on different Illumina platforms. We analyzed the two cell lines separately [[15](#_ENREF_15)].

**GSE19043** comprises 21 microarrays, analysis of DiFi cell line and GTL-16 cell line, treated with Gefitinib (and other agents, not used here). We separately analyzed the two cell types [[16](#_ENREF_16)].

**GSE23175** comprises 48 microarrays, analysis of MCF10-A and MCF10HER cell lines treated with Gefitinib from 0 to 45 hrs. We separately analyzed the sensitive and resistant cell types, but combined all the treated time points [[17](#_ENREF_17)].

**GSE34557** comprises 12 microarrays, analysis of primary human epidermal keratinocytes treated or not with BMP2 +/- AG1478. We separately analyzed the BMP2-treated and untreated cultures [[18](#_ENREF_18)].

**References to this supplement**:

1. Nagashima T, Ushikoshi-Nakayama R, Suenaga A, Ide K, Yumoto N, et al. (2009) Mutation of epidermal growth factor receptor is associated with MIG6 expression. Febs J 276: 5239-5251. doi: 5210.1111/j.1742-4658.2009.07220.x. Epub 02009 Aug 07210.

2. Liu L, Greger J, Shi H, Liu Y, Greshock J, et al. (2009) Novel mechanism of lapatinib resistance in HER2-positive breast tumor cells: activation of AXL. Cancer Res 69: 6871-6878. doi: 6810.1158/0008-5472.CAN-6808-4490. Epub 2009 Aug 6811.

3. Albitar L, Pickett G, Morgan M, Wilken JA, Maihle NJ, et al. (2010) EGFR isoforms and gene regulation in human endometrial cancer cells. Mol Cancer 9:166.: 10.1186/1476-4598-1189-1166.

4. Ware KE, Marshall ME, Heasley LR, Marek L, Hinz TK, et al. (2010) Rapidly acquired resistance to EGFR tyrosine kinase inhibitors in NSCLC cell lines through de-repression of FGFR2 and FGFR3 expression. PLoS One 5: e14117. doi: 14110.11371/journal.pone.0014117.

5. Lee MJ, Ye AS, Gardino AK, Heijink AM, Sorger PK, et al. (2012) Sequential application of anticancer drugs enhances cell death by rewiring apoptotic signaling networks. Cell 149: 780-794. doi: 710.1016/j.cell.2012.1003.1031.

6. Fertig EJ, Ren Q, Cheng H, Hatakeyama H, Dicker AP, et al. (2012) Gene expression signatures modulated by epidermal growth factor receptor activation and their relationship to cetuximab resistance in head and neck squamous cell carcinoma. BMC Genomics 13: 160.

7. Massarweh S, Tham YL, Huang J, Sexton K, Weiss H, et al. (2011) A phase II neoadjuvant trial of anastrozole, fulvestrant, and gefitinib in patients with newly diagnosed estrogen receptor positive breast cancer. Breast Cancer Res Treat 129: 819-827. doi: 810.1007/s10549-10011-11679-10548. Epub 12011 Jul 10527.

8. Creighton CJ, Massarweh S, Huang S, Tsimelzon A, Hilsenbeck SG, et al. (2008) Development of resistance to targeted therapies transforms the clinically associated molecular profile subtype of breast tumor xenografts. Cancer Res 68: 7493-7501. doi: 7410.1158/0008-5472.CAN-7408-1404.

9. Kojic N, Chung E, Kho AT, Park JA, Huang A, et al. (2010) An EGFR autocrine loop encodes a slow-reacting but dominant mode of mechanotransduction in a polarized epithelium. Faseb J 24: 1604-1615. doi: 1610.1096/fj.1609-145367. Epub 142010 Jan 145367.

10. Chan G, Nogalski MT, Yurochko AD (2009) Activation of EGFR on monocytes is required for human cytomegalovirus entry and mediates cellular motility. Proc Natl Acad Sci U S A 106: 22369-22374. doi: 22310.21073/pnas.0908787106. Epub 0908782009 Dec 0908787111.

11. Carey LA, Rugo HS, Marcom PK, Mayer EL, Esteva FJ, et al. (2012) TBCRC 001: randomized phase II study of cetuximab in combination with carboplatin in stage IV triple-negative breast cancer. J Clin Oncol 30: 2615-2623. doi: 2610.1200/JCO.2010.2634.5579. Epub 2012 Jun 2614.

12. Nakachi I, Naoki K, Soejima K, Kawada I, Watanabe H, et al. (2010) The combination of multiple receptor tyrosine kinase inhibitor and mammalian target of rapamycin inhibitor overcomes erlotinib resistance in lung cancer cell lines through c-Met inhibition. Mol Cancer Res 8: 1142-1151. doi: 1110.1158/1541-7786.MCR-1109-0388. Epub 2010 Jul 1120.

13. Zhang Z, Lee JC, Lin L, Olivas V, Au V, et al. (2012) Activation of the AXL kinase causes resistance to EGFR-targeted therapy in lung cancer. Nat Genet 44: 852-860. doi: 810.1038/ng.2330.

14. Komurov K, Tseng JT, Muller M, Seviour EG, Moss TJ, et al. (2012) The glucose-deprivation network counteracts lapatinib-induced toxicity in resistant ErbB2-positive breast cancer cells. Mol Syst Biol 8:596.: 10.1038/msb.2012.1025.

15. Kalinowski FC, Giles KM, Candy PA, Ali A, Ganda C, et al. (2012) Regulation of epidermal growth factor receptor signaling and erlotinib sensitivity in head and neck cancer cells by miR-7. PLoS One 7: e47067. doi: 47010.41371/journal.pone.0047067. Epub 0042012 Oct 0047024.

16. Bertotti A, Burbridge MF, Gastaldi S, Galimi F, Torti D, et al. (2009) Only a subset of Met-activated pathways are required to sustain oncogene addiction. Sci Signal 2: ra80. doi: 10.1126/scisignal.2000643.

17. Giordano CR, Mueller KL, Terlecky LJ, Krentz KA, Bollig-Fischer A, et al. (2012) A targeted enzyme approach to sensitization of tyrosine kinase inhibitor-resistant breast cancer cells. Exp Cell Res 318: 2014-2021. doi: 2010.1016/j.yexcr.2012.2006.2001. Epub 2012 Jun 2018.

18. Mulder KW, Wang X, Escriu C, Ito Y, Schwarz RF, et al. (2012) Diverse epigenetic strategies interact to control epidermal differentiation. Nat Cell Biol 14: 753-763. doi: 710.1038/ncb2520.
